# Suppurtive Inflammation of the Middle Ear

**Published:** 1880-08

**Authors:** Jno. H. Curry

**Affiliations:** Toledo, O.


### SUPPURTIVE INFLAMMATION OF THE MIDDLE EAR.

By JNO. H. CURRY, M.D., Toledo, O.

It is not the design of the paper to discuss this subject fully or in detail. This would draw too heavily upon your time and perhaps your patience. But merely to attract the attention of any who have been hitherto inattentive to this subject. The text books treat of it thoroughly and exhaustively and attach to it the importance which it deserves. Notwithstanding this, the affection is often practically ignored. The olden time spirit has not yet been entirely eradicated, and we occasionally hear of cases where suppuration was patiently awaited, nay, even desired, and then allowed to continue, "Because it is unsafe to stop the discharge, as it might go to some other part." Further, the people have not yet been made to comprehend that an ear-ache is not a trivial matter, but that it may lead to very serious results, and that the physician should be called to attend to and look after it with the same promptness as he would be in a case of peritonitis or pneumonia. Purulent inflammation of the middle ear is a grave affection, both in its primary and secondary effects. A mere glance at the anatomy of the tympanum, its relation and proximity to other important parts, will convince any one of this, who will give it the least thought. The intimate connection of the cavity with the mastoid cells, shows how readily inflammation in it might be conveyed to them, thus inducing a dangerous and annoying complication. The thin, bony partition which separates it from the dura mater, and this wall sometimes has fissures in it, shows how easily meningitis might be caused, by an extension of inflammation. The lower wall separates the cavity from the jugular vein, and anatomists tell us that the bone is sometimes absent, leaving the partition membranous. In such condition, or when the bone becomes carious, we may fear an implication of the vein and phlebitis; since, in suppuration of the tympanum, the floor is usually covered with pus. The facial nerve through a portion of its course is separated from the drum cavity by a thin partition of bone, and we might, with good reason, fear that it would become involved by disease in the cavity, and produce temporary, or permanent, facial paralysis. In front of the promontory the inner wall of the tympanum consists of a very thin plate of bone, separating this cavity from the carotid artery. This plate is pierced by many minute openings for vessels and nerves. Should this wall be broken down by disease, there is nothing to prevent ulceration of the carotid, and attendant hemorrhage.

From the anatomy of the cavity we must logically infer that the above cited pathological conditions would be very likely produced by certain forms of disease within it, and an abundant clinical experience proves that this conclusion is correct. In the light of these facts, which are readily obtained by any one who will take the trouble to search for them, it is surprising how often this affection is wholly ignored. We have to deal with an affection which not only threatens the destruction of the sense of hearing but also the life of the patient. There is no doubt that many persons have died from affections directly caused and kept up by purulent inflammation of the middle ear, and that too when the ear trouble was not noted and did not receive any treatment whatever. I have seen pyæmia, for instance, kept up for weeks by suppuration in the middle ear, and the ear trouble received no attention. In children who cannot locate accurately the seat of the pain, we may get convulsions and treat the child for brain affection, until apprised of the real location of the disease, by a discharge from the ear. If the ear disease is still neglected and a meningitis actually induced, it is not surprising that the first diagnosis is thought to have been correct, and the ear disease considered of no moment and treated accordingly. Purulent inflammation of the middle ear is a frequent complication in the exanthemata, and I am constrained to believe, does not receive the attention which its importance demands. It is painful and annoying, it draws upon the nervous and vital forces of the already enfeebled patient. It may destroy the physiological function of the organ. It is a source from which the blood may become poisoned, or by the implication of contiguous structures become even more threatening to the life of the patient than the systemic disease. It should, therefore, receive prompt and immediate treatment. Mr. Hinton, of London, expresses the opinion that in scarlet fever, for instance, if the ear complication received proper treatment, we would often save the lives of patients.

*Symptomatology.*—The affection is usually excruciatingly painful. We are all familiar with the quaint description of the pain in gout, in which we are told that after giving so many turns of a vise, when one's thumb is in it, we must give one more turn, and this gives a faithful idea of the pain. I question if this pain, or any other, is more unbearable than that which is sometimes produced by pus confined in the tympanic cavity, and forcibly pressing the membrana tympani for an outlet. This pain is usually one of the most prominent symptoms. It is located deep in the ear, and radiates over the side of the face, also causes headache. If we find that the auricle and outer parts of the ear can be freely handled, and are not at all sensitive, we may exclude them. If, however, these parts are also involved, or there are abscesses in the external auditory canal, the diagnosis is more difficult. Where we find that the fauces were first noticeably affected, and the pain radiated towards the ear, we may generally conclude that the middle ear and enstachian tube are involved, the hearing is usually impaired, and the patient complains of tinnitus aurium, and sometimes vertigo. The positive evidences are gained only by an inspection of the membrana tympani. In the beginning we find this congested about the periphery and along the mallus handle. Later the drum membrane becomes boggy, swollen, and all traces of the normal membrane are gone. If there is a perforation we get a discharge of pus from the auditory canal, and are usually able to see the opening in the membrane. If, failing in this we get the perforation whistle upon inflation, we may feel certain there is a hole through the drum head. Ordinarily when the membrana tympani gives way and the pus is allowed to make its way out through the auditory canal, the pain ceases. If it does not, or when having subsided it returns, we may suspect that other parts have become involved. When the parts behind the auricle become swollen, red and tender to the touch, we may be sure of it, and direct our attention to the mastoid process. A brief report of a few cases will perhaps best illustrate the clinical features, treatment, etc.

In 1879, Mr. —— consulted me with reference to a terrible pain and noise in his right ear. The pain had been present continuously for several days, and prevented him from resting by day or sleeping at night. He located it deep in the ear, from this point it radiated over the side of the face and into the head; he felt nervous and exhausted. The hearing was greatly impaired. The membrana tympani was greatly swollen, and red throughout its entire extent; no trace of the light spot, malleus handle, or the usual distinguishing features of the membrane could be discovered. At one point it bulged outwards into the auditory canal, and appeared thinned. The external auditory canal about the drum head, was also inflamed and sensitive. Throat was slightly sore and fauces inflamed. Eustachian tube permeable. I considered it certain that the drum was full of pus, and that it would perforate the drum head, if left to itself. A paracentesis was done, and there followed the withdrawal of the knife a considerable quantity of bloody purulent matter. The ear was douched with warm water several times per day. Once per day the Eustachian tube and drum were gently inflated with the catheter, or Politzer's method, and the auditory canal and parts about the puncture carefully cleansed. Patient was ordered to stay at home and keep quiet. After the sensitiveness had passsd away, the syringe was substituted for the douche, and patient was given a solution of zinci sul. grs. iii to the ℥ i of water, to be warmed and put into the ear twice per day, after syringing. On my part the daily inflation and cleasing was kept up, and a solution of argenti nitras carefully brushed about the edges of the incision, which had been kept open by the discharge. In about twenty days the discharge had ceased, and the hole in the membrane was firmly healed. Hearing not measurably impaired.

There was a typical case of acute purulent inflammation of the middle ear; where the pain was intense and the drum head resisted obstinately the destructiveness of the affection and the pressure of the pus seeking an escape. The result was a rapid return to a normal condition. Had the disease been allowed to run its course, the great probabilities are, that a large portion of the membrana tympani would have been swept away, or even the ossicles with the membrane, and a chronic suppuration established which might have been a life-long affair, to say nothing of the possible complications. It will be observed that I punctured the bulging drum head. The object was to limit, if possible, the injury to the membrane and secure the escape of the pus, thus removing the pressure. Had the rupture been allowed to take place spontaneously, we could not be sure as to its location or extent. I believe this little operation, when properly employed, gives us an advantage and control which we could not otherwise exercise. I know it has, like some other medical procedures, become fashionable and may sometimes be resorted to when there is no absolute demand for it. I do not advocate its use except when the conditions indicate that the drum head will otherwise burst, or when there is fluid or diseased products in the drum, which cannot otherwise escape, and may prove injurious if allowed to remain. Under these circumstances, I believe we shirk our duty if we fail to employ it.

Case II.—R. S., aged seven, was brought to my office April 27, 1880, with the following history: A few days previously right ear began to ache violently, and kept up for about twenty minutes. His mother steamed his ear and immediately the pain ceased and a discharge began.

*Status præsens.*—Pus in the auditory canal, membrana tympani swollen and red, and a small perforation in lower front quadrant. The ear was inflated and cleansed, at first daily, and an application of argenti nitras made to the perforation and inflamed membrane occasionally. Ordered frequent syringing with warm water, and twice per day an application of solution sulphate of zinc, warm. The suppuration ceased in about one week, and on May 27 perforation was firmly healed and the hearing fully established. Fauces were swollen and tonsils enlarged. For this a gargle of solution of potassæ chloras was used.

Case III.—Mr. J. T. consulted me February 27, 1880. He stated that one week previously he took cold, and his ears began to ache violently. Four days afterwards the right ear began to discharge. The next day the left ear commenced discharging and the pain ceased. The pus flowed freely from the ears. The hearing was much impaired and the ears sensitive when handled.

*Status præsens.*—Both auditory canals filled with pus. Right ear drum greatly swollen, red and perforated; can't make out the full size of the perforation on account of the swollen and changed condition of the parts. Left membrani tympani much swollen and inflamed, perforated at lower and posterior quadrant, pulsation at point of perforation. The air whistles through distinctly when the drums are inflated. Ordered frequent syringing with warm water, and twice per day an instillation of astringent solution. Inflated the drums through the Eustachian tubes once per day; cleansed the parts carefully and brushed them with a solution of nitrate of silver. Throat was inflamed. For this, ordered gargle. Within a few weeks discharge had ceased, and on March 31, both drums were firmly healed and hearing good.

Case IV.—C. H., August 23, 1879, stated that two weeks previously his right ear began to ache; had run a little, but the aching still kept up.

*Status præsens.*—Ear very sensitive; cannot handle the auricle without producing pain; auditory canal swollen and nearly shut its entire length; pus deep in the auditory canal; drum membrane cannot be seen. A point in the auditory canal looks as if it contains pus. The suspected point was lanced, and I ordered the ear douched frequently with warm water and leeches applied. When the swelling of the canal was allayed sufficiently, the membrana tympani was found to be perforated. After the tenderness and swelling had subsided, the syringe was substituted for the douche, and the treatment was the same as of the previous cases. The discharge ceased and perforation healed, leaving the hearing good. In this case I believe the disease began in the external auditory canal and traveled inward.

Case V.—The past spring was called to see a little son of Mr. A. He was convalescing from an attack of measles. A few days previous to my visit his ears began to ache. When I saw him he seemed in constant agony. Uttered piercing screams, and cried incessantly "my ears! my ears!" Found the throat inflamed and tonsils enlarged. Both drum-heads were swollen, red and bulging. No perforation. Ordered the ears douched frequently with hot water and leeches applied; gargle for the throat and an opiate internally. Pain subsided and ears grew rapidly better. Parents then thought they could take care of them. A week or so afterwards was again sent for, and found the little patient worse than at first visit. One drum-head was now perforated. After a brief treatment the affection subsided and perforation healed. The other drum-head did not burst.

I have taken these cases just as they came, without any special selection, further than to show different stages of the affection. It will be observed that in some of the cases the membranæ tympani were perforated when they came under observation. In another a paracentesis was done, while in the last case one of the drums did not rupture. In another case there was extensive trouble in the auditory canal. All of these cases recovered. By recovery is meant a stoppage of the discharge and a healing of the perforation. This cannot always be done. Cases occur where we cannot heal the perforation; these are, fortunately, exceptions to a general rule. I think statistics will show that 80 per cent. can be cured. With regard to the application of solutions of nitrate of silver, and astringents, care and discretion must be used. If they are made too early they will almost certainly increase the excitement and pain. The question may arise, how would such cases terminate if left to themselves? Undoubtedly there are cases where the perforation heals without any treatment, but the very best we can expect from the great majority is a chronic suppuration, which may go on for life, and a loss of practical hearing. The following case will answer this question in one direction: November 25, 1875, was called to see a boy about three years old. He was having scarlet fever when his ears became involved. Found the throat inflamed and swollen, tonsils enlarged. Both ears were suppurating freely and both drums were perforated. The post-auricular region was swollen and tender on pressure. After a few days' treatment the mastoid symptoms subsided. The patient was so situated that I could not visit him and the parents did not think it best to bring him to me. The danger of mastoiditis was pointed out, etc. Still they undertook to watch the case and carry out the treatment. I next saw the case March 22, 1876. Both ears were suppurating, and the child was suffering severe pain, especially in the right ear. The drum and inner part of the ext. auditory canal were nearly filled with polypi. The tissue behind the auricle was swollen almost even with its edge, very tender to the touch and fluctuation well marked. It was evident that the mastoid bone was diseased and pus was burrowing about in the soft tissues. Child looked haggard, was feverish and broken down. A deep incision was made down to the bone, which let out a quantity of pus. An inspection of the bone revealed a fistulous opening. This was probed, carefully cleansed and enlarged. The polypi about the remains of the drum-head and in the tympanum were removed, after which water injected into the ear passed freely out through the opening, in the mastoid process, and vice versa. From this time treatment was kept up, cleasing applications to the tympanum, removal, and destruction of the polypi, which persistently formed, and inflation through the Eustachian tubes. The general health was carefully looked after. The child improved in its general condition. Left ear stopped discharging and drum healed, but the right continued to discharge until December. It then became apparent that a sequestrum had formed in the mastoid which would have to be removed. This was done December 22. It consisted of quite a large piece of bone which contained a portion of the mastoid cells. The condition now improved rapidly, discharge ceased, and the opening over the mastoid process closed. This was the course of one case which was not neglected, but treated inefficiently. I think all will agree that the case is suggestive and well calculated to establish respect for earache, and instruct us as to the possible consequences of an inefficiently treated or neglected purulent inflammation of the middle ear. This subject is too large to discuss fully in a paper which must necessarily be limited, and which is already of sufficient length. I wish, however, to call attention briefly to the diseases which we frequently get as secondary results—chronic suppuration, with a partial or practically total loss of the hearing; changes in the tympanum with adhesion of the various structures, impairing the hearing for life; inflammation and necrosis of the mastoid bone, with exfoliation; meningitis, cerebritis, and cerebral abscess; pyæmia and septicamia; disease and caries of the tables of the calvarium; disease of the facial nerve with facial paralysis; thrombosis of the sinuses of the brain; ulceration of the carotid artery and its attendant consequences; disease of the jugular vein and phlebitis. This is certainly a formidable list of formidable affections, and ought to impress us with a wholesome awe of any affection which stands to them in relation of cause. These affections are frequently reported where their dependence upon middle ear trouble was clearly recognized. The literature of the subject furnishes a long list; they are not possibilities but probabilities, and no patient is ever out of danger from them so long as he is the victim of suppurating middle ear. These facts, gentlemen, I deem sufficient to sustain the declaration made at the beginning of the paper, viz: That purulent inflammation of the middle ear is a grave affection, and should receive early and prompt attention.

